# RBM47/SNHG5/FOXO3 axis activates autophagy and inhibits cell proliferation in papillary thyroid carcinoma

**DOI:** 10.1038/s41419-022-04728-6

**Published:** 2022-03-25

**Authors:** Yuan Qin, Wei Sun, Zhihong Wang, Wenwu Dong, Liang He, Ting Zhang, Chengzhou Lv, Hao Zhang

**Affiliations:** Department of Thyroid Surgery, The First Hospital of China Medical University, 155 Nanjing Bei Street, Shenyang, Liaoning Province 110001 People’s Republic of China

**Keywords:** Oncogenes, Head and neck cancer

## Abstract

Papillary thyroid carcinoma (PTC) is the main type of thyroid carcinoma. Despite the good prognosis, some PTC patients may deteriorate into more aggressive diseases, leading to poor survival. Molecular technology has been increasingly used in the diagnosis and treatment of thyroid carcinoma. In this study, we identified that RNA Binding Motif Protein 47 (RBM47) was downregulated in PTC tissues and cells, and overexpression of RBM47 could activate autophagy and inhibit proliferation in PTC cells. RBM47 promotes but can not bind directly to Forkhead Box O3 (FOXO3). FOXO3 activates Autophagy Related Gene 3 (ATG3), ATG5, and RBM47 to form a loop and promote autophagy. RBM47 can bind directly to and stabilized lncRNA Small Nucleolar RNA Host Gene 5 (SNHG5) to inhibit PTC cells proliferation and activate autophagy in vitro and in vivo. SNHG5 inhibits ubiquitination and degradation of FOXO3 by recruiting Ubiquitin Specific Peptidase 21 (USP21), then promotes the translocation of FOXO3 from cytoplasm to nucleus. Our study revealed the regulatory mechanism of RBM47/SNHG5/FOXO3 axis on cell proliferation and autophagy in PTC, which may provide valuable insight for the treatment of PTC.

## Introduction

Thyroid carcinoma is the most common endocrine-related malignant tumor [1]. Most of this growth is attributed to papillary thyroid carcinoma (PTC), which generally have a good prognosis and can be efficiently cured by conventional therapies [2]. Despite the good prognosis, some patients with PTC may deteriorate into more aggressive disease such as nerve and vascular invasion, local and distant metastasis, and recurrence, which will less respond adequately to conventional therapies, ultimately leading to poor patient survival [3–5]. Therefore, studies on molecular regulation mechanism and new therapeutic targets for thyroid cancer are in desperate need.

RNA Binding Proteins (RBPs) control RNA splicing, localization, stability and translation efficiency through direct binding, thereby regulating tumor biological processes [6]. For instance, TAF15 can stabilize LINC00665 to impede the malignant biological behaviors of glioma cells [7]. RBM47, plays an important role in regulating mRNA transcription, RNA splicing, and RNA transportation [8]. RBM47 has been considered as a tumor suppressor as it suppressed the progression of breast cancer, colon cancer, and lung cancer [9–12]. However, the expression level and biological function of RBM47 in thyroid carcinoma remain unclear.

Long non-coding RNAs (lncRNAs) are non-coding RNAs with a transcript of more than 200 nt. Increasing studies have demonstrated that the abnormal expression of lncRNAs exhibits tumor-promotional or tumor-suppressive effects in various cancers [13–15]. For example, lncRNA GAS8-AS1 enhances cell autophagy and hampers cell proliferation in thyroid carcinoma [16]. SNHG5, one of the well-defined cytoplasmic lncRNAs, also called U50HG, is located in human chromosome 6q14.3 with a length of 524 bp. Aberrant expression of SNHG5 has been reported in several human cancers including hepatocellular carcinoma, colorectal cancer, and breast cancer [17–19]. Nonetheless, the functional role and molecular mechanism of SNHG5 in thyroid carcinoma have not been explored so far.

FOXO3 belongs to a large family of Forkhead Box (FOX) transcription factors [20, 21]. The abnormal activation of FOXO3 has been widely studied in the occurrence and development of cancer [22–24]. FOXO3 is low-expressed in thyroid carcinoma and plays a proapoptotic role during tumorigenesis by regulating apoptosis-related genes [25–27]. Autophagy is a highly conserved cellular process, which degrades bulk cytoplasmic materials under starvation to maintain cellular homeostasis. Cytoplasmic proteins and organelles are sequestered by the growing double membrane, forming autophagosomal vesicles, which will fuse with lysosomes to degrade the contents [28]. Studies showed that FOXO3 could promote autophagy by transcriptionally activating autophagy-related genes, which indicated that FOXO3 can be used as a marker of autophagy [29–31].

In this study, we investigated that RBM47 can inhibit proliferation and promote autophagy by binding and stabilizing SNHG5. SNHG5 could recruit USP21, which interacted with FOXO3 to inhibit its ubiquitination and further promote its nuclear translocation. FOXO3 activates autophagy by promoting ATG3 and ATG5 transcription and activates RBM47 transcription to form a positive feedback loop. These results will provide new molecular mechanisms for thyroid carcinoma development and new insights for thyroid carcinoma treatment.

## Methods

### Sample collection

One hundred pairs of PTC tissues and corresponding adjacent non-cancerous tissues were obtained from patients undergoing thyroidectomy at the First Hospital of China Medical University from 2014 to 2018. All samples were immediately dissected, placed on ice, snap-frozen in liquid nitrogen, then stored at −80 °C until use. The patient tissue samples were confirmed by histopathological examination to be PTC tissues and adjacent non-cancerous tissues. The collected clinicopathological characteristics included age, gender, extrathyroidal extension, TNM stage (AJCC 8th), Lymph node metastasis (LNM), multicentricity, tumor size, and Hashimoto thyroiditis. None of the patients had received preoperative local or systemic treatment. All procedures involving human participants in the study were in accordance with the ethical standards of the Research Ethics Committee of The First Hospital of China Medical University as well as the 1964 Helsinki declaration and its later amendments. All PTC patients involved in this study have signed informed consent so that their samples can be obtained.

### Cell culture and cell transfection

Human PTC cell lines (TPC1, BCPAP, K1, IHH4) and a normal thyroid follicular cell line (Nthy-ori 3–1) were used in our study. The cell source and culturing methods have been described previously [32].

To knock down RBM47, SNHG5, USP21, or FOXO3, short hairpin RNAs (shRNAs) targeting RBM47 or SNHG5 (sh-RBM47 or sh-SNHG5) and short interfering RNAs (siRNAs) targeting USP21 or FOXO3 (si-USP21 or si-FOXO3) were transfected into thyroid carcinoma cells. Non-targeting shRNAs or siRNAs were used as negative controls (sh-NC or si-NC). To overexpress RBM47, SNHG5, USP21, or FOXO3, we cloned complete coding sequences of these genes into lentivirus or pcDNA3.1 vectors, respectively (Lv-RBM47, Lv-SNHG5, pcDNA3.1/USP21, or pcDNA3.1/FOXO3). Empty lentivirus or pcDNA3.1 vectors were used as negative controls (Lv-NC or pcDNA3.1). The siRNAs and the si-NC were made in GenePharma (Suzhou, China). The expression plasmids and lentivirus vectors were made in Obio Technology (Shanghai, China). According to the manufacturer’s proposals, siRNAs or plasmids were transfected into TPC1 and BCPAP cells with Lipofectamine 3000 Reagents (Invitrogen, USA). The siRNA sequences are available in Table S3.

### Cell Counting Kit-8 (CCK-8) assay

Three thousands of TPC1 and BCPAP cells were seeded in each well of 96-well plates in a final volume of 100 µl, respectively. Proliferation was evaluated at 0, 24, 48, and 72 h after transfection. The cells were incubated for 3 h at 37 °C after 10 µl CCK-8 solvent was supplemented to each well. The number of viable cells was calculated based on the absorbance at 450 nm.

### Total RNA isolation and quantitative real-time PCR (qRT-PCR)

Total RNA was isolated from frozen tissue and cell samples by RNAiso (Takara, Dalian, China). A reverse transcription kit (RR036A, Takara, Shiga, Japan) was used to transcribe total RNA and produce complementary DNA. For the analysis of gene expression, qRT-PCR was performed using SYBR Premix Ex Taq II (Takara) and the LightCycler 480 system (Roche, Indianapolis, IN, USA). The relative expression levels were calculated using the 2^−ΔCt^ method (Ct, cycle threshold). ΔCt indicates the difference in the Ct value between a target gene and the endogenous reference. GAPDH was used as the internal control. Each PCR was performed in triplicate to verify the stability and repeatability of the results. The primer sequences are available in Table S3.

### Western blotting (WB)

Total proteins were extracted from the cells by a lysis buffer, and the protein concentration was detected using a bicinchoninic acid protein assay kit (Beyotime, China). Proteins were fractionated by 10% or 12% sodium dodecyl sulfate-polyacrylamide gel electrophoresis. The separated proteins were transferred to polyvinylidene fluoride membranes (Bio-Rad, Hercules, CA, USA). The membranes were blocked in 5% skim milk at room temperature for 2 h, and then incubated with primary antibodies at 4°C overnight. Next, the blotted membranes were incubated with horseradish peroxidase-conjugated anti-rabbit immunoglobulin G (1:20,000) secondary antibody at room temperature for 2 h. The proteins were visualized using an enhanced chemiluminescence detection system. Information on the primary antibodies is available in Table S4.

### Immunohistochemical (IHC) analysis

We obtained fresh tumor tissues and the corresponding non-cancerous tissues from 46 PTC patients to construct the tissue microarray (TMA). The TMA sections were baked at 65 °C for 30 min, then deparaffinized and rehydrated with xylene and alcohol gradients, respectively. The endogenous peroxidase activity was blocked with 3% H_2_O_2_. Next, the sections were treated with citrate buffer, microwave‐heated for 20 min, then incubated with anti-RBM47 and anti-FOXO3 antibodies overnight at 4 °C. The IHC staining score was evaluated using the semi‐quantitative Remmele scoring system. Information on the primary antibodies is available in Table S4.

### Immunofluorescence (IF) assay

Cells (1 × 10^5^ cells/well) were seeded in six-well plates. The cells were washed with phosphate-buffered saline after transfection, fixed with 4% polyformaldehyde, infiltrated with 0.2% Triton X-100, cultured in 3% hydrogen peroxide without light, blocked with 5% bovine serum albumin (BSA), and then incubated with primary antibodies against LC3 (1:500; Abcam, US) and FOXO3 (1:200; Proteintech, US) solutions at 4 °C overnight. The next day, after removing the primary antibodies solutions, the transfected cells were incubated with secondary antibody solutions (1:100; ZSGB-BIO, Beijing, China) for 2 h, then stained with diaminophenylindole (DAPI, Beyotime) and visualized by a confocal microscope (Leica). Information on the primary antibodies is available in Table S4.

### Transmission electron microscopy (TEM)

Cells were collected with cell scraper, centrifuged, and fixed in 2.5% glutaraldehyde 0.1 M sodium cacodylate buffer. Then, the cells were dehydrated in a gradient of 50–100% ethanol and embedded in araldite. Ultrathin sections (50–60 nm) were stained with uranyl acetate and lead citrate. A JEM-1400 transmission electron microscope was applied under 80 kV with ×20,000 magnification.

### Subcellular fractionation

The cytoplasmic and nuclear fractions of TPC1 or BCPAP cells were isolated using the Cytoplasmic and Nuclear RNA Purification Kit (Norgen Biotek, Ontario, Canada). The cell suspension was centrifuged and precipitated. Then lysis buffer J was added for centrifugation. The remaining supernatant contained the cytoplasmic RNA, and the precipitate was the nuclear RNA. The supernatant was transferred to an RNase-free tube. Buffer SK and 96–100% ethanol were added to the cytoplasmic RNA and nuclear RNA, respectively. The mixture was applied to a spin column comprised of a collection tube and centrifuged, the flow-through was discarded, and the spin column was reassembled with its collection tube. Subsequently, wash solution A was applied to the column for centrifugation, and then the flow-through was discarded. The previous steps were repeated to wash the column three times. Elution buffer E (50 µl) was added to the column for centrifugation, and the remaining supernatant contained the cytoplasmic RNA and the nuclear RNA. The purified RNA samples could be stored at –20 °C for a few days and at –70 °C for long-term storage.

### Fluorescence in situ hybridization (FISH)

Probes were used to identify SNHG5 rearrangement in TPC1 and BCPAP cells (green-labeled, Boster, Wuhan, China). The cells were seeded on 24-well slides and fixed with 4% paraformaldehyde (POM) for 30 min. The pepsin was digested with 3% citric acid for 2 min, then fixed in 1% POM for 30 min. The pre-hybridizing liquid was added at 38–42 °C for 2–4 h, and then the hybridizing liquid was added at 38–42 °C overnight. The next day, after washing out the buffer, the cells were treated with blocking solution for 30 min, biotinylated rat anti-digoxin for 2 h, SABC (Strept Avidin-Biotin Complex) for 30 min, and biotinylated peroxidase for 30 min. All fluorescent images were captured by a confocal microscope (Leica, Wetzlar, Germany).

### Bioinformatics analysis

Through ENCORI website (http://starbase.sysu.edu.cn/), SNHG5 was predicted to bind to RBM47. FOXO3 was predicted to be a potential upstream transcriptional regulator of RBM47, ATG3, and ATG5 by using online databases UCSC (http://genome.ucsc.edu/) and JASPAR (http://jaspar.genereg.net/).

### RNA immunoprecipitation (RIP) assay

RIP assay was carried out with the EZ-Magna RNA-binding protein immunoprecipitation kit (Millipore, USA) in this study. The lysates of TPC1 cells were incubated with RIP buffer, magnetic beads, human anti-RBM47 (Abcam, UK) or anti-USP21 (Proteintech Group), and negative control normal mouse IgG (Millipore, USA). Next, the mixture was incubated with proteinase K. Then, the immunoprecipitated RNA was purified, and the RNA concentration and quality were assessed by a NanoDrop spectrophotometer (Thermo Scientific). Finally, qRT-PCR was used to analyze the purified RNA with specific primers to illustrate the existence of the binding targets.

### RNA stability evaluation

De novo synthesis of RNA was inhibited by adding actinomycin D (MCE, China) to cell culture medium. Total RNA was isolated at different time points (0, 4, 8, and 12 h), respectively, and tested by qRT-PCR. The half-life of RNA was defined as the time when the RNA level dropped to 50% of the baseline level.

### Reporter vector construction and luciferase reporter assay

To construct the reporter vectors, the promoter regions of ATG3, ATG5, and RBM47 were cloned and amplified as wild type (WT) vectors, and the promoter regions of them with the predicted FOXO3 binding sequences depletion were cloned and amplified as mutant type (Mut) vectors. TPC1 cells were seeded in 96-well plates and co-transfected with FOXO3 overexpression vectors or empty vectors and whole or deleted promoter regions vectors using Lipofectamine 3000. After 48 h, the luciferase activity was evaluated by a dual luciferase reporter system (Promega, Beijing, China).

### Chromatin immunoprecipitation (ChIP) assay

To verify the predicted FOXO3 binding sites on the promoter regions of ATG3, ATG5, and RBM47 respectively, ChIP assay was performed with the simple ChIP Enzymatic Chromatin IP Kit (Cell signaling Technology, Danvers, MA, USA). TPC1 cells were cross-linked with 1% formaldehyde for 10 min, then glycine was added for incubation for 5 min at room temperature to terminate the crosslinking. Next, the cells were collected, lysed in 1% PMFS lysis buffer, and incubated with micrococcal nuclease for 20 min at 37 °C. Then, the samples were incubated with FOXO3 antibody or IgG antibody (negative control) at 4 °C with gentle shaking overnight. In addition, 5 mol/l NaCl and proteinase K were used to de-crosslink at 65 °C for 2 h. Finally, immunoprecipitated DNA was purified and amplified with primers, and then isolated and visualized by 3% agarose gel. Information on the primers is shown in Table S3.

### Xenograft tumor model

The xenograft tumor models were constructed in BALB/c nude mice (4–5 weeks old) purchased from Beijing Vital River Laboratory Animal Technology Co., Ltd., (Beijing, China). The tumor volumes were estimated by tumor length and width every 3 days. One month later, the mice were sacrificed, and the tumors were excised and weighed. All animal studies were conducted in accordance with the principles and procedures outlined in the guidelines of the Institutional Animal Care and Use Committee (IACUC) of China Medical University. (IACUC approval number: KT2020136)

### Statistical analysis

All statistical analyses were performed with SPSS 22.0 (IBM, Chicago, IL, USA) and GraphPad Prism 8.0 (GraphPad Software, La Jolla, CA, USA). The relative expression of RBM47, SNHG5, FOXO3, ATG3, and ATG5 in the PTC and adjacent non-cancerous tissues was analyzed by the Wilcoxon signed-rank test. The correlation between clinicopathological characteristics with the expression of RBM47 and SNHG5 was examined by the chi-square test. Data are presented as the means ± standard deviation (SD), and statistical analyses were performed by Student’s t-test or analysis of variance. The differences were deemed statistically significant with *p < 0.05 or **p < 0.01.

## Results

### RBM47 activates autophagy and inhibits proliferation in PTC cells

**Fig. 1:**
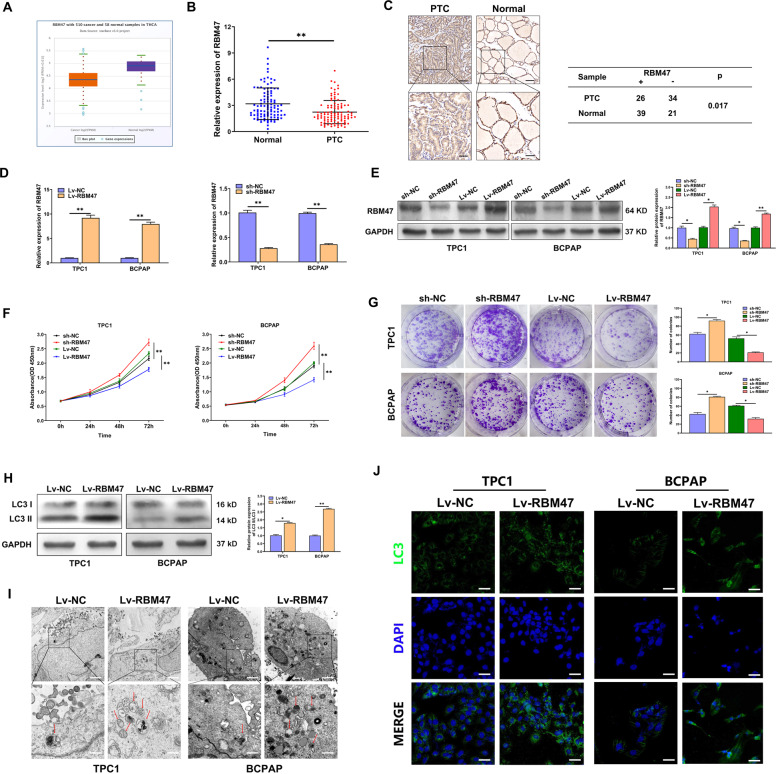
RBM47 inhibits proliferation and activates autophagy in PTC cells. **A** RBM47 expression in PTC tissues and normal tissues from TCGA database. **B** The relative mRNA expression of RBM47 in 100 paired PTC tissues and adjacent non-cancerous tissues. **C** Representative RBM47 IHC staining of PTC tissues and matched adjacent non-cancerous tissues. Scale bar = 50 μm (Upper) and 20 μm (Below). **D**, **E** The transfection efficiency of sh-RBM47 and Lv-RBM47 assessed by qRT-PCR and WB in TPC-1 and BCPAP cells. **F**, **G** Proliferation of RBM47-silenced or -overexpressed cells evaluated by CCK-8 assay and plate colony assay. **H**–**J** LC3 II/LC3 I ratio detected by WB, LC3 puncta evaluated by IF and autophagosomes shown by TEM (red arrow) in PTC cells with RBM47 upregulation. Scale bar in TEM = 5 μm (Upper) and 1 μm (Below), Scale bar in IF = 20 μm. Statistical differences were analyzed using the independent samples t-test; data are shown as the mean ± standard error of the mean based on three independent experiments. *p < 0.05, **p < 0.01.

The RNA-seq data from the Cancer Genome Atlas (TCGA) and the qRT-PCR results of the 100 paired PTC tissues and corresponding adjacent non-cancerous tissues indicated that the expression of RBM47 significantly decreased in PTC tissues (Fig. 1A, B). According to the IHC results of 60 paired PTC tissues and matched adjacent non-cancerous tissues, the protein expression of RBM47 was also significantly downregulated in PTC tissues (Fig. 1C). We also found that low RBM47 expression was positively associated with bigger tumor size and higher TNM stage of PTC patients (Table S1). For further analysis, CCK-8 assay and plate colony were applied to assess the effect of RBM47 on the proliferation of PTC cells. As expected, RBM47 upregulation resulted in significant inhibition on cell proliferation, while RBM47 knockdown promoted the proliferative rates of PTC cells (Fig. 1D–G). Autophagy plays an important role in regulating the proliferation of tumor cells. To investigate whether autophagy affects the proliferation inhibition caused by RBM47, WB, IF and TEM analyses were conducted to evaluate autophagy flux. WB analysis demonstrated that the PTC cells with RBM47 upregulation exhibited a significantly higher LC3 II/LC3 I ratio compared with the control (Fig. 1H). Bafilomycin A1, an inhibitor of autophagosome-lysosome fusion, markedly blocked RBM47-induced autophagy flux and further increased the accumulation of LC3-II, indicating an accelerated conversion from LC3-I to LC3-II (Fig. S1A). RBM47 overexpression resulted in more autophagosomes and LC3 stained punctate aggregates, which were observed by TEM and IF (Fig. 1I–J). In addition, we also observed that RBM47 mediated proliferation inhibition could be partially restored by autophagy inhibitor, Bafilomycin A1 in PTC cells (Fig. S1B). According to the above results, RBM47 was downregulated in both PTC tissues and cell lines, and inhibited proliferation by activating autophagy of PTC cells.

### RBM47 activates autophagy by regulating FOXO3

**Fig. 2:**
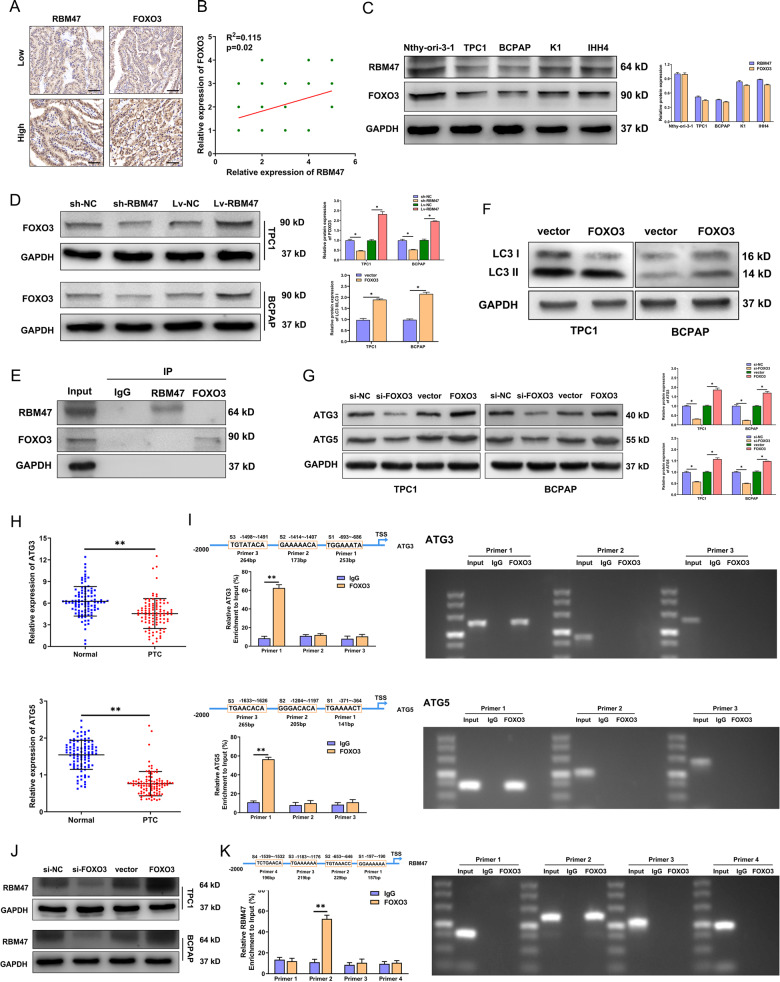
RBM47 activates autophagy by regulating FOXO3. **A** Representative RBM47 and FOXO3 IHC staining of PTC tissues and their correlation. Scale bar = 20 μm. **B** The correlation of RBM47 and FOXO3 in PTC tissues measured by IHC. **C** The protein expression of RBM47 and FOXO3 in PTC cells and normal thyroid cells measured by WB. **D** The protein levels of FOXO3 were detected after RBM47 knockdown or upregulation. **E** TPC1 cell lysates were subjected to Co-IP with anti-RBM47 or anti-FOXO3 antibody, respectively, and normal rabbit IgG was the control, followed by WB using the indicated antibodies. **F** LC3 II/LC3 I ratio detected by WB in PTC cells with FOXO3 upregulation. **G** The protein levels of ATG3 and ATG5 detected by WB in PTC cells with FOXO3 knockdown or upregulation. **H** The relative mRNA expression of ATG3 and ATG5 in 100 paired PTC tissues and adjacent non-cancerous tissues. **I** ChIP analysis of FOXO3 binding sites in ATG3 or ATG5 promoter regions in TPC1 cells. IgG was used as the negative control. **J** The protein levels of RBM47 in PTC cells with FOXO3 knockdown or upregulation. **K** ChIP analysis of FOXO3 binding sites in RBM47 promoter region in TPC1 cells. IgG was used as the negative control. Statistical differences were analyzed using the independent samples t-test; data are shown as the mean ± standard error of the mean based on three independent experiments. *p < 0.05, **p < 0.01.

FOXO3 is a classic transcription factor that can transcriptionally activate autophagy-related genes to promote autophagy. However, whether RBM47 activates autophagy by regulating FOXO3 remains unclear. Similar to RBM47, FOXO3 was also downregulated in PTC tissues (Fig. 2A). Subsequently, IHC analyses and western blot showed a positive correlation between RBM47 and FOXO3 in PTC tissues and cell lines (Fig. 2B, C). We speculated there was a regulatory relationship between RBM47 and FOXO3. In exploring the regulatory network, we observed that FOXO3 protein levels decreased in RBM47-silenced cells and increased upon RBM47 overexpression, while there was no significant difference in FOXO3 mRNA level (Figs. 2D and S2A). Subsequently, IP experiments confirmed that RBM47 could not directly bind to FOXO3 (Fig. 2E). We not only identified that FOXO3 activated autophagy to verify the results of previous studies, but also found that FOXO3 inhibited cell proliferation, which was regulated by autophagy (Figs. 2F and S2B). Then we further explore the mechanism by which FOXO3 activates autophagy in PTC cells. ATGs play a key role in the multistep catabolic processes of autophagy. We confirmed that the expression of ATG3 and ATG5 decreased in FOXO3 downregulated cells and increased in FOXO3 up-regulated PTC cells (Figs. 2G and S2C). We also found that ATG3 and ATG5 expression significantly decreased in PTC tissues (Figs. 2H and S2D). Through UCSC and JASPAR, it was predicted that FOXO3 could bind to ATG3 or ATG5 promoter with 3 binding sites, respectively. Luciferase reporter gene and ChIP assay all revealed that FOXO3 interacted with the promoter of ATG3 or ATG5 (Figs. 2I and S2E). Additionally, we also found that FOXO3 activated the transcription of RBM47 to form a feedback loop (Figs. 2J, K and S2F, G). These results indicated that RBM47/FOXO3 positive feedback loop activated autophagy by promoting ATG3 and ATG5. However, the mechanism of RBM47 regulating FOXO3 remains unclear.

### RBM47 stabilized SNHG5 to inhibit proliferation and activate autophagy in PTC cells

**Fig. 3:**
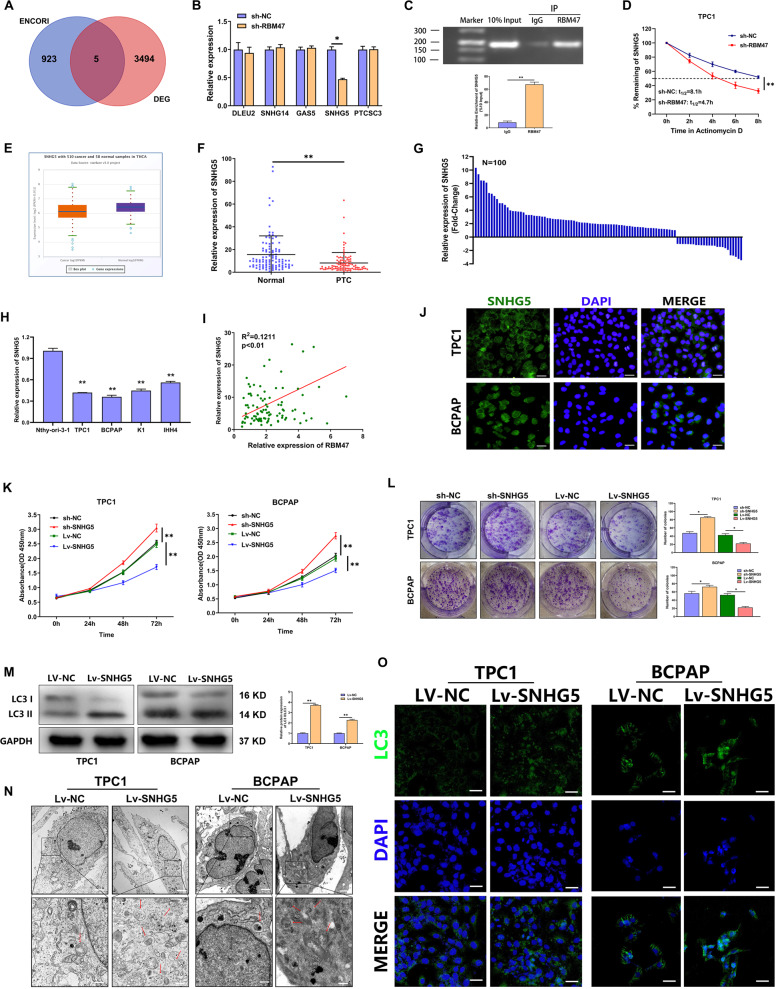
RBM47 stabilized SNHG5 to inhibit proliferation and activate autophagy in PTC cells. **A** The lncRNAs that potentially bind to RBM47 predicted by bioinformatics software. **B** The relative mRNA expression of related lncRNAs detected by qRT-PCR in TPC1 cells with RBM47 knockdown. **C** The enrichment of SNHG5 expression in anti-RBM47 precipitates shown by RIP assay. **D** The half-life of SNHG5 in TPC1 cells treated with RBM47 knockdown. **E** SNHG5 expression in PTC tissues and normal tissues from TCGA database. **F** The relative mRNA expression of SNHG5 in 100 paired PTC tissues and adjacent non-cancerous tissues. **G** The fold change of SNHG5 expression in 100 PTC tissues. **H** The relative mRNA expression of SNHG5 in PTC cells and normal thyroid cells measured by qRT-PCR. **I** The correlation between RBM47 and SNHG5 in PTC tissues. **J** The localization of GAS8-AS1 in cytoplasm verified by FISH. Scale bar = 20 μm. **K**, **L** Proliferation of SNHG5-silenced or -overexpressed cells evaluated by CCK-8 assay and plate colony assay. **M**–**O** LC3 II/LC3 I ratio detected by WB, LC3 puncta evaluated by IF and autophagosomes shown by TEM (red arrow) in PTC cells with SNHG5 upregulation. Scale bar in TEM = 5 μm (Upper) and 1 μm (Below), scale bar in IF = 20 μm. Statistical differences were analyzed using the independent samples t-test; data are shown as the mean ± standard error of the mean based on three independent experiments. *p < 0.05, **p < 0.01.

As a multifunctional RBP, RBM47 could bind mRNA and alter the abundance of its target mRNAs. To explore the downstream genes regulated by RBM47, ENCORI database was used to predict the lncRNAs binding to RBM47. A total of 5 candidate lncRNAs were obtained by taking the intersection with the lncRNAs differentially expressed in the PTC microarray [33] (Fig. 3A). QRT-PCR analysis showed that SNHG5 was the only positively regulated by RBM47 in PTC cells (Figs. 3B and S3A). Subsequently, a RIP assay was performed and demonstrated that RBM47 could bind to SNHG5 (Fig. 3C). We also found that the half-life of SNHG5 was significantly shortened in the RBM47 knockdown group compared with that in the NC group (Figs. 3D and S3B). To clarify the role of SNHG5 in PTC, SNHG5 expression was tested by qRT-PCR in PTC tissues and cell lines (TPC1, BCPAP, K1, and IHH4). The results indicated that SNHG5 significantly decreased in PTC tissues and cell lines (Fig. 3E–H). Correlation analysis showed a positive correlation between RBM47 and SNHG5 in PTC tissue (Fig. 3I). Moreover, low expression of SNHG5 was associated with an increased risk of LNM and larger tumor size in patients with PTC (Table S2). Subcellular fractionation and RNA FISH analyses were conducted and found that SNHG5 was mainly distributed in the cytoplasm (Figs. 3J and S3C). Finally, we explored the function of SNHG5 on PTC cell and confirmed that SNHG5 inhibited PTC cell proliferation but activated autophagy (Figs. 3K–O and S3D, E). We also observed that blocking autophagy could partially restore the proliferation inhibition caused by SNHG5 (Fig. S3F). The above results indicated that RBM47 stabilized SNHG5 to inhibit proliferation and activate autophagy in PTC cells.

### RBM47 regulates PTC cell proliferation and autophagy via SNHG5 in vitro and in vivo

**Fig. 4:**
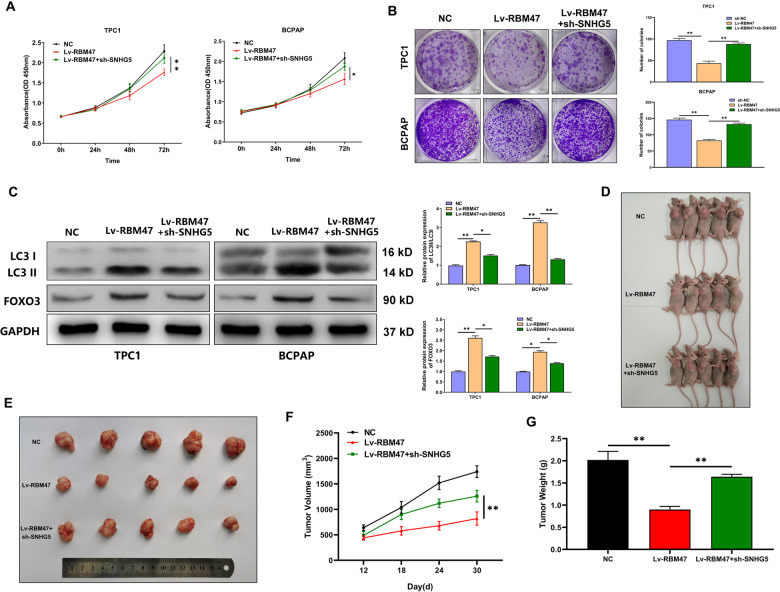
RBM47 regulates PTC cell proliferation and autophagy via SNHG5 in vitro and in vivo. **A**, **B** Proliferation of PTC cells transfected with sh-NC, Lv-RBM47, or Lv-RBM47 + sh-SNHG5 evaluated by CCK-8 assay and plate colony assay. **C** LC3 II/LC3 I ratio and FOXO3 protein level detected by WB in PTC cells transfected with sh-NC, Lv-RBM47, or Lv-RBM47 + sh-SNHG5. **D**, **E** The subcutaneous implant mouse models inoculated with sh-NC, Lv-RBM47, or Lv-RBM47 + sh-SNHG5 TPC1 cells (*n* = 5). **F**, **G** Volumes and weights of harvested tumors in both groups. Scale bar = 20 μm. Statistical differences were analyzed using the independent samples t-test; data are shown as the mean ± standard error of the mean based on three independent experiments. *p < 0.05, **p < 0.01.

To verify whether RBM47 regulates PTC cell proliferation via SNHG5, we designed and performed some restoration assays in PTC cells. As illustrated in Fig. 4A, B, CCK-8 and plate colony assays delineated that SNHG5 downregulation offset the suppressed cell proliferation in RBM47 upregulated cells. WB implied that the increased LC3 II/LC3 I ratio in RBM47 upregulated cells was rescued by SNHG5 downregulation. What’s more, SNHG5 downregulation resisted the increase of FOXO3 expression caused by RBM47 overexpression (Fig. 4C). To clarify whether RBM47 influences tumor growth via SNHG5 in vivo, TPC1 cells stably transfected with NC, Lv-RBM47, or Lv-RBM47 + sh-SNHG5 were injected into the backs of nude mice. The growth of subcutaneous tumors was observed every 6 days from the 12th day after injection. The tumor volume was calculated based on the long diameter and short diameter measured by Vernier caliper. The results showed that the size, volume, and weight of xenograft tumors in the Lv-RBM47 group were remarkably lower than those in the NC group, whereas sh-SNHG5 co-transfection could rescue the inhibitory effect of RBM47 overexpression on tumor growth (Fig. 4D–G). The above results suggested that RBM47 regulates PTC cell proliferation and autophagy via SNHG5 in vitro and in vivo.

### SNHG5 regulates ubiquitination and promotes expression of FOXO3 in PTC cells

**Fig. 5:**
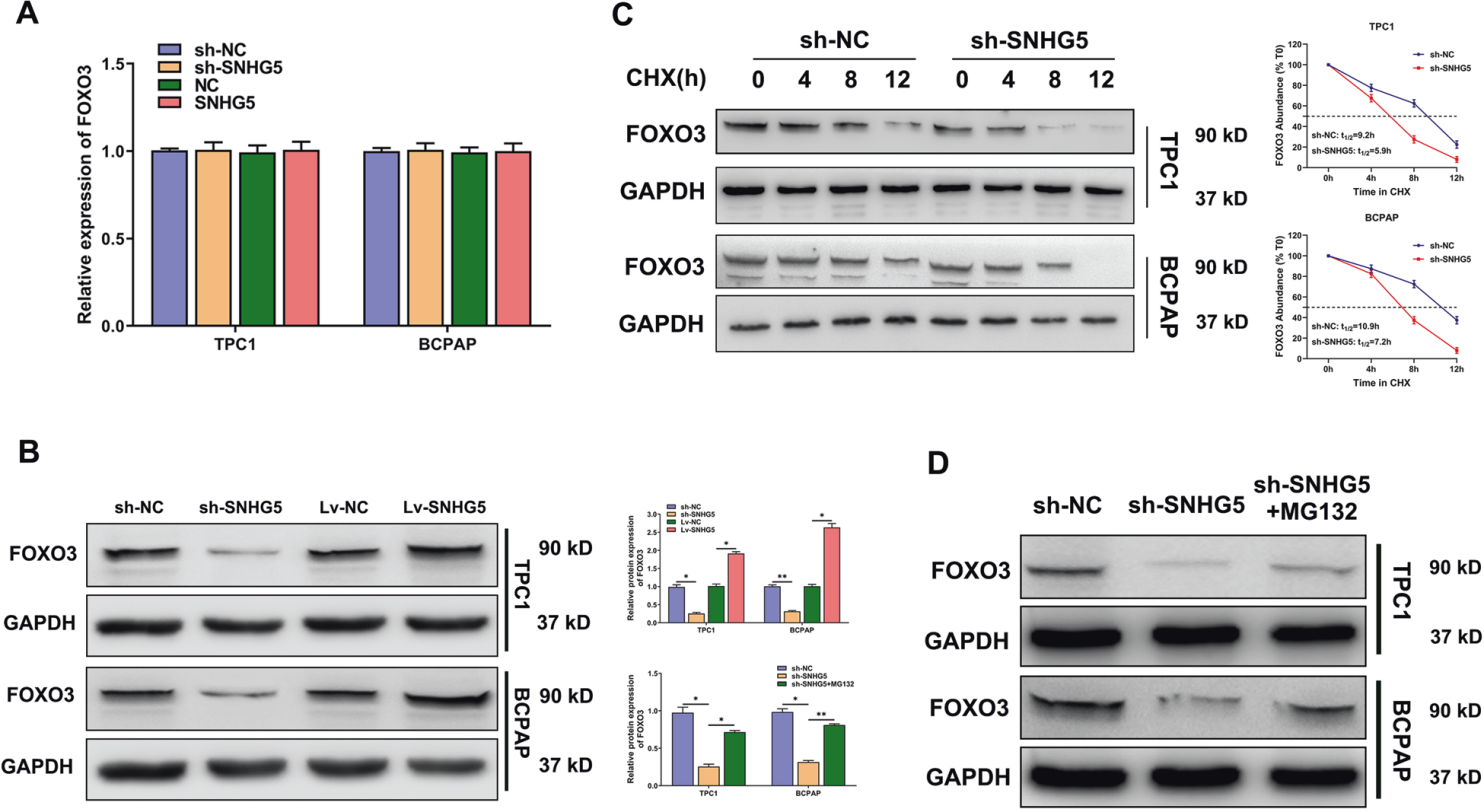
SNHG5 regulates ubiquitination and promotes expression of FOXO3 in PTC cells. **A**, **B** The relative mRNA and protein levels of FOXO3 after SNHG5 knockdown or upregulation. **C** PTC cells were transfected with sh-SNHG5 and sh-NC for 72 h, then treated with 100 ng/mL CHX. The samples were obtained every 4 h in the 12 h after transfection. **D** PTC cells were transfected with sh-SNHG5 for 72 h, then treated with 10 mM MG132 for 12 h.

Next, the regulatory mechanism of SNHG5 on FOXO3 was explored. WB results revealed that FOXO3 protein levels decreased in SNHG5-silenced cells and increased in SNHG5 overexpressed cells, while there was no significant difference in FOXO3 mRNA level. This indicated that SNHG5 could regulate FOXO3 expression at the post-transcriptional level (Fig. 5A, B). To evaluate the impact of SNHG5 on FOXO3 stability, we used WB to assess the half-time of FOXO3 in SNHG5-silenced PTC cells, which were treated with cycloheximide (CHX) to block protein translation. FOXO3 abundance fell more rapidly in SNHG5 depletion cells than in control cells (Fig. 5C). To determine whether the effect of SNHG5 knockdown on FOXO3 is mediated by proteasomal degradation, PTC cells transfected with sh-SNHG5 were treated with the proteasome inhibitor MG132. WB analysis confirmed that FOXO3 destabilization resulting from SNHG5 knockdown could be partially rescued through proteasome inhibition (Fig. 5D). Altogether, these results indicated that SNHG5 regulated FOXO3 proteasomal-mediated degradation.

### SNHG5 regulates FOXO3 expression by recruiting USP21

**Fig. 6:**
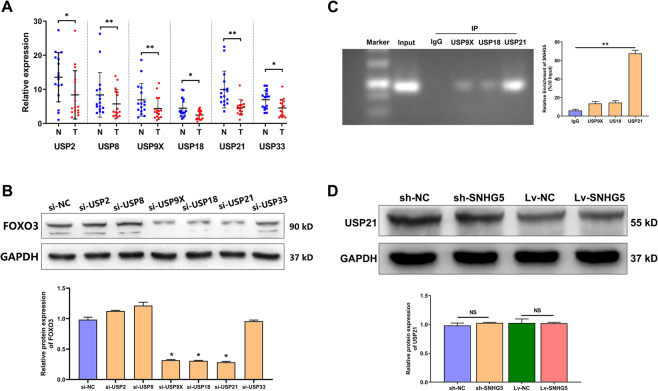
SNHG5 regulates FOXO3 expression by recruiting USP21. **A** The relative mRNA expression of potential USP-family DUBs in 24 paired PTC tissues and adjacent non-cancerous tissues. **B** FOXO3 abundance assessed by WB in TPC1 cells transfected with siRNAs targeting respective DUBs. **C** The enrichment of SNHG5 in anti-USP21 precipitates shown in RIP assay. **D** The protein level of USP21 detected in PTC cells with SNHG5 knockdown or upregulation.

Ubiquitin-specific proteases (USPs) belong to the deubiquitinase (DUB) family, which can inhibit the ubiquitination of target genes by removing their ubiquitin markers. To discover the USPs that directly affect FOXO3 abundance, 6 USPs were selected as candidates, including USP2, USP8, USP9X, USP18, USP21, and USP33, which were differentially expressed in 24 paired PTC tissues and adjacent non-cancerous tissues (Fig. 6A). WB analysis revealed that USP9X, USP18, and USP21 knockdown reduced FOXO3 levels (Fig. 6B). RIP assays further confirmed that only USP21 can bind with SNHG5 (Fig. 6C). Additionally, we found that SNHG5 could not regulate the expression of USP21 (Fig. 6D). In general, these findings suggested that SNHG5 might act as a scaffold binding with USP21 to regulate FOXO3 expression.

### USP21 inhibits FOXO3 ubiquitination and promotes its nuclear translocation

**Fig. 7:**
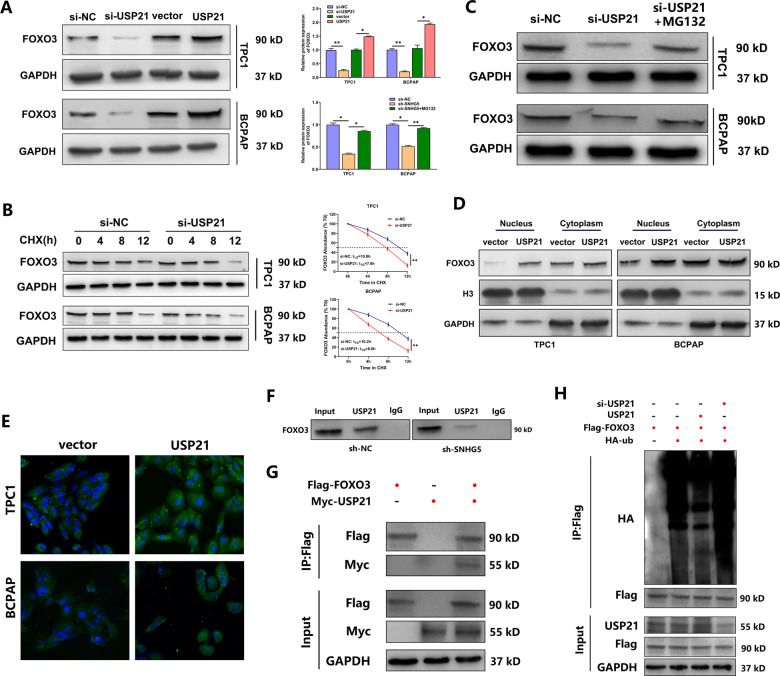
USP21 inhibits FOXO3 ubiquitination and promotes its nuclear translocation. **A** The protein level of FOXO3 in PTC cells with USP21 knockdown or upregulation. **B** PTC cells were transfected with si-USP21 or si-NC for 48 h, then treated with 100 ng/mL CHX. Samples were taken every 4 h for 12 h. **C** PTC cells were transfected with si-USP21 for 48 h, then treated with 10 mM MG132 for 12 h. **D** The cytoplasmic and nuclear levels of FOXO3 in PTC cells transfected with vector and pcDNA3.1/USP21. **E** The effect of USP21 on FOXO3 translocation determined by IF assay. Scale bar = 20 μm. **F** SNHG5 knockdown decreased the interaction between USP21 and FOXO3 in TPC1 cells. **G** The protein complexes immunopurified with anti-Flag or anti-Myc and analyzed by WB in TPC1 cells co-expressed with Flag-FOXO3 and Myc-USP2. **H** Immunoprecipitation with anti-Flag antibodies in TPC1 cells co-transfected with HA-ub, Flag-FOXO3, si-USP21, and pcDNA3.1/USP21. The indicated proteins were probed by WB analysis.

Then we evaluated the impact of USP21 on FOXO3 stability. FOXO3 abundance decreased in USP21-silenced cells and increased in USP21 overexpressed cells (Fig. 7A). Subsequently, we used WB to assess the half-life of FOXO3 in USP21-silenced PTC cells treated with CHX. FOXO3 abundance fell more rapidly in USP21-silenced cells than in control (Fig. 7B). We also confirmed that FOXO3 destabilization resulting from USP21 knockdown was partially rescued by the proteasome inhibitior MG132 (Fig. 7C). We further investigated whether USP21 affected FOXO3 subcellular localization. Remarkable enhancement of FOXO3 was found in USP21 overexpressed PTC cells via WB and IF assay, and the overexpressed FOXO3 was localized not only in the cytoplasm but also in the nucleus, particularly the latter. This indicated the accumulated FOXO3 was transported to nucleus (Fig. 7D, E). Finally, IP assay confirmed that SNHG5 increased the binding of USP21 to FOXO3, and USP21 can further deubiquitinated and stabilized FOXO3 (Fig. 7F–H). These data demonstrated that USP21 inhibited FOXO3 ubiquitination by interacting with it.

## Discussion

The incidence of PTC has been rapidly increasing over the past few decades. Although the prognosis of patients with PTC is generally good, tumor invasiveness and metastasis are the major risk factors that lead to poor prognosis [2, 34]. Accumulating studies have revealed that RBPs regulate the expression of thousands of transcripts, some of which have been reported to be involved in various cancers. Discovery of the driver RBPs is critical for precision oncology [35, 36]. As a RBP, RBM47 inhibits lung cancer growth and metastasis by inhibiting Nuclear Factor Erythroid-2 related Factor 2 (NRF2) activity and modulating AXIN1 mRNA stability [10, 12]. Autophagy is a highly conserved process of cell self-degradation, which plays a vital role in cell survival under stress conditions [37]. However, the effect of RBM47 on autophagy remains unknown. In this study, RBM47 was significantly downregulated in thyroid carcinoma, and overexpression of RBM47 inhibited proliferation and activated autophagy in PTC cells.

FOXO3 is a classical and well-studied transcription factor that can activate ATGs to promote autophagy in a variety of tumors. For example, FOXO3-induced transcription of ATG7 promote autophagy in non-small-cell lung cancer. However, a similar role in thyroid carcinoma has not been established. To further investigate the target genes of FOXO3, we focused on several ATGs, and found that only the expression of ATG3 and ATG5 significantly decreased with FOXO3 knockdown at both the mRNA and protein levels. UCSC and JASPAR predicted that there were 3 binding sites between ATG3 or ATG5 promoter and FOXO3, which was further verified in luciferase reporter and CHIP assays. This indicated that FOXO3 activated autophagy through ATG3 and ATG5. In exploring the regulatory network, we observed that FOXO3 protein level decreased in RBM47-silenced cells, while there was no significant difference at the mRNA level. This indicated that RBM47 could regulate FOXO3 at the post-transcriptional level. However, we occasionally found that RBM47 was decreased by FOXO3 knockdown and increased by FOXO3 overexpression in qRT-PCR and WB assays. Then, luciferase reporter and ChIP assays showed that FOXO3 could activate RBM47 transcription to form a positive feedback loop.

IP experiments have indicated that RBM47 can not bind directly to FOXO3. Therefore, the regulation of FOXO3 by RBM47 aroused our interest. As a multifunctional RBP, RBM47 could bind mRNA and alter the abundance of its target mRNAs. Therefore, we speculated whether RBM47 could function by binding lncRNA. LncRNA SNHG5 has been elucidated to exhibit oncogenic property in various cancers [38–41]. In this study, SNHG5 was downregulated in PTC, and overexpression of SNHG5 inhibited PTC cells proliferation, but activated autophagy. Additionally, we also found that RBM47 can bind directly to and stabilized SNHG5 to inhibit PTC cells proliferation and activate autophagy in vitro and in vivo.

FOXO3 is regulated by various post-translational modifications, which influence the subcellular localization, protein stability, and transcriptional activity of FOXO3 [20]. Ubiquitinated FOXO3 will be degraded by the proteasome. FOXO3 proteins accumulate and shuttle from cytoplasm to nucleus under the action of the proteasome inhibitor MG132. The FOXO3 accumulated in nucleus can directly bind to a series of gene promoters and regulate their transcription [25]. There may be a potential strategy to activate FOXO3 by targeting FOXO3 ubiquitinases. DUBs remove ubiquitin from modified proteins to inhibit ubiquitination. Therefore, we tried to identify deubiquitinases that regulate FOXO3 stability and protect FOXO3 from degradation. USP21 is a member of the USP-family DUBs, which contain 56 members with a highly conserved USP domain and a catalytic triad [42]. USP21 has been involved in transcriptional regulation by interacting with transcriptional factors NANOG, GATA3, and GLI1 as well as histone H2A [43]. In this study, USP21 knockdown shortened the half-life of FOXO3 in the presence of the translational inhibitor CHX. MG132 treatment restored the decreased FOXO3 expression resulting from USP21 knockdown. Overexpression of USP21 drastically decreased the ubiquitination and promoted the nuclear transfer of FOXO3. Additionally, RIP assays confirmed the binding between SNHG5 and USP21. All the results indicated that USP21 recruited by SNHG5 interacted with FOXO3 to inhibit its ubiquitination and promote its nuclear translocation.

## Conclusions

**Fig. 8:**
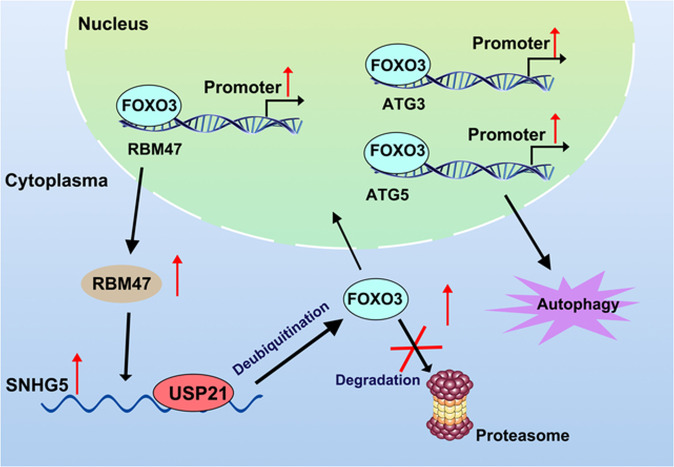
The regulation mechanism of RBM47/SNHG5/USP21/FOXO3 axis in PTC cells. In brief, the upregulation of RBM47 inhibits PTC cell proliferation and active autophagy by binding to SNHG5. SNHG5 can recruited USP21, which interacts with FOXO3 to inhibit its ubiquitination and further promote its nuclear translocation. FOXO3 activates autophagy by promoting ATG3 and ATG5 transcription and activates RBM47 transcription to form a positive feedback loop.

Based on the above results, we conclude that upregulation of RBM47 inhibits PTC cell proliferation and active autophagy by binding to SNHG5. SNHG5 can recruit USP21, which interacts with FOXO3 to inhibit its ubiquitination and further promote its nuclear translocation. FOXO3 activates autophagy by promoting ATG3 and ATG5 transcription and activates RBM47 transcription to form a positive feedback loop. In general, our study revealed the regulation mechanism of RBM47/SNHG5/FOXO3 axis on PTC cell proliferation and autophagy, which may provide valuable insight for the treatment strategy of PTC (Fig. 8).

## Supplementary information

Supplementary western blot

Supplemental Figure legends

Supplementary Table 1

Supplementary Table 2

Supplementary Table 3

Supplementary Table 4

Supplemental Figure1

Supplemental Figure2

Supplemental Figure3

Reproducibility checklist

## Data Availability

The data that support the findings of this study are available from the corresponding author upon reasonable request.
