# Supplementary figures and images for: RBM47/SNHG5/FOXO3 axis activates autophagy and inhibits cell proliferation in papillary thyroid carcinoma
Source: Cell Death Dis. 2022 Mar 25;13(3):270. doi: 10.1038/s41419-022-04728-6 (PMC8956740; doi:10.1038/s41419-022-04728-6)

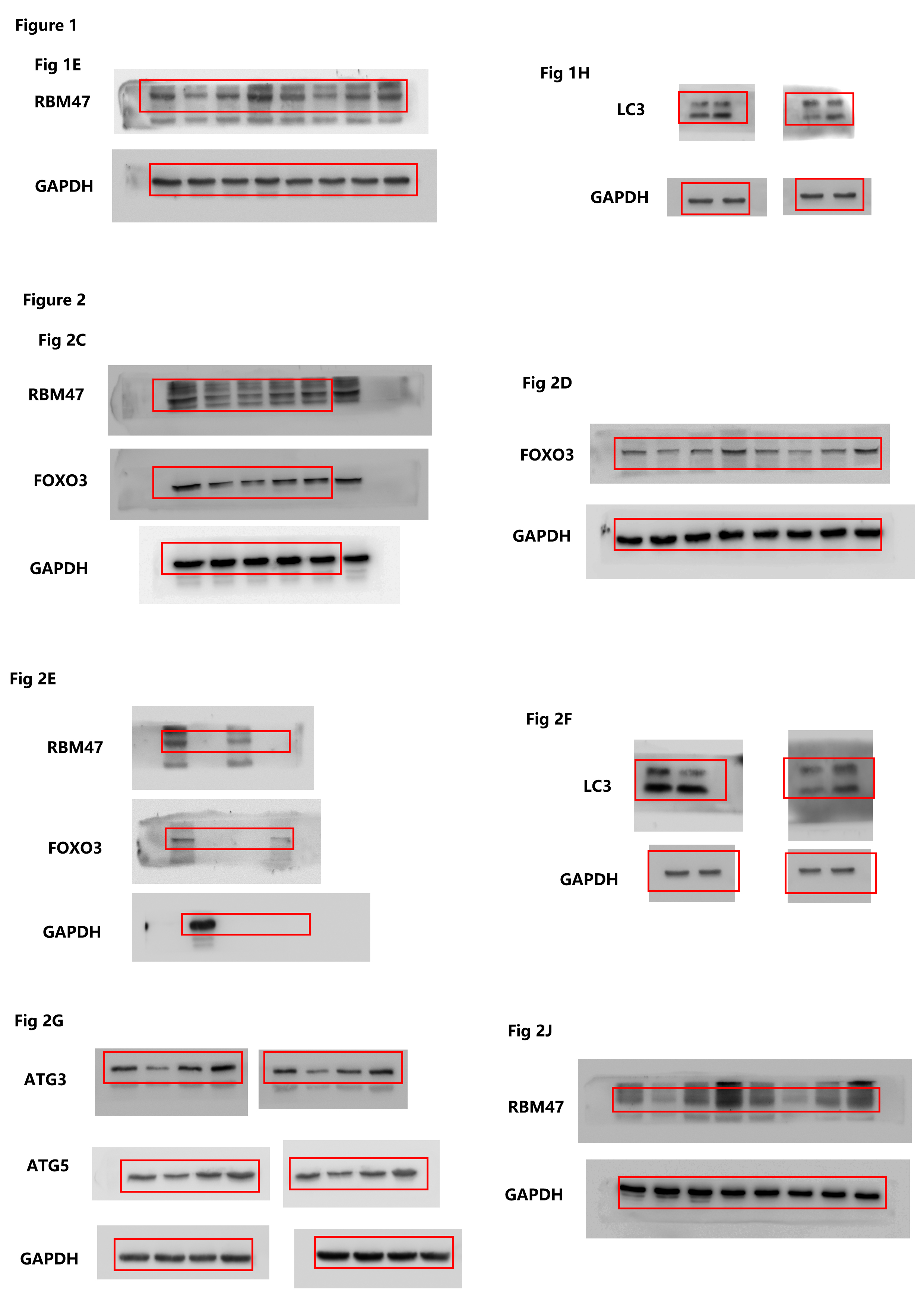

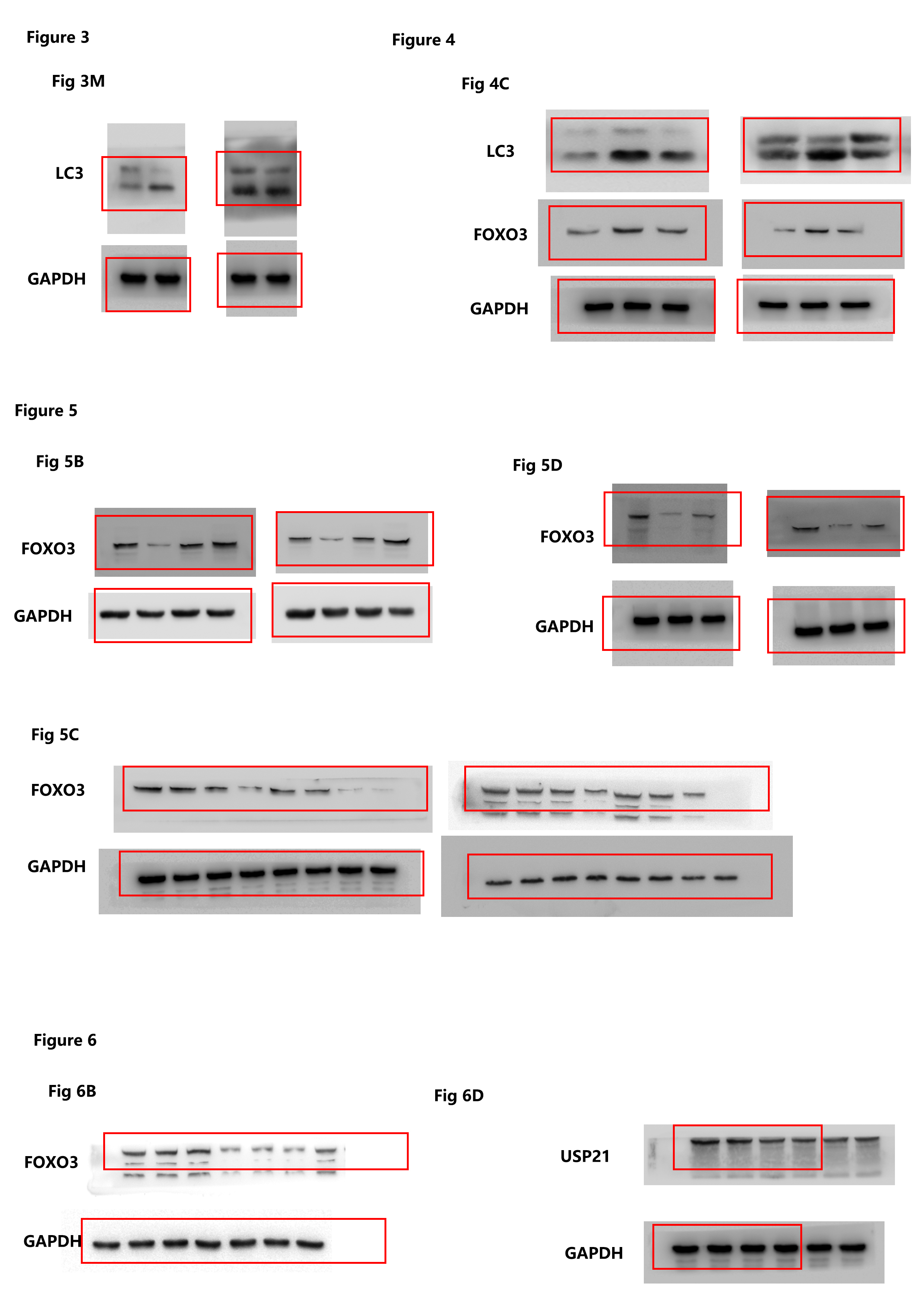

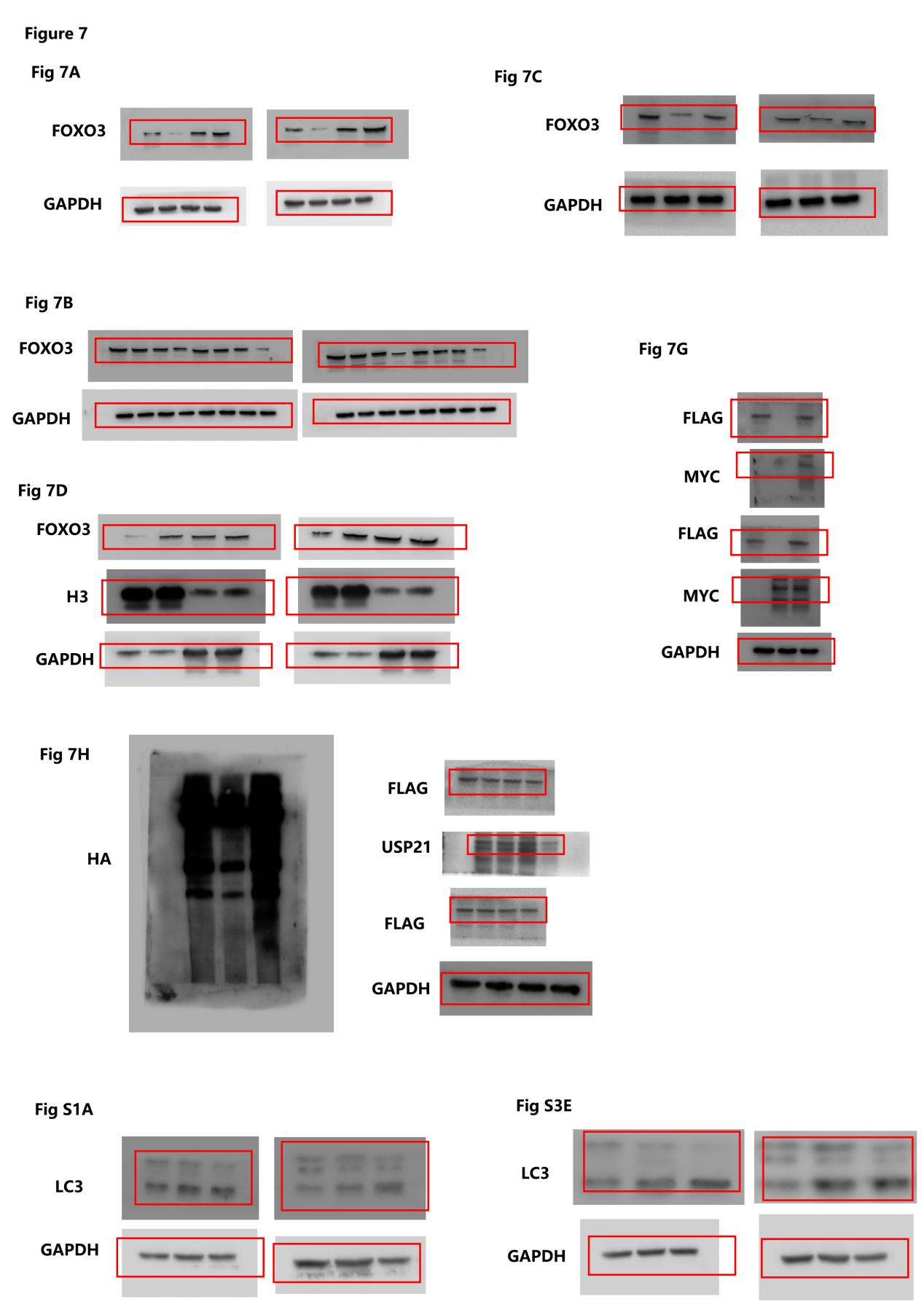

Supplement: Supplementary file 1 — Supplementary western blot [file 41419_2022_4728_MOESM1_ESM.docx]

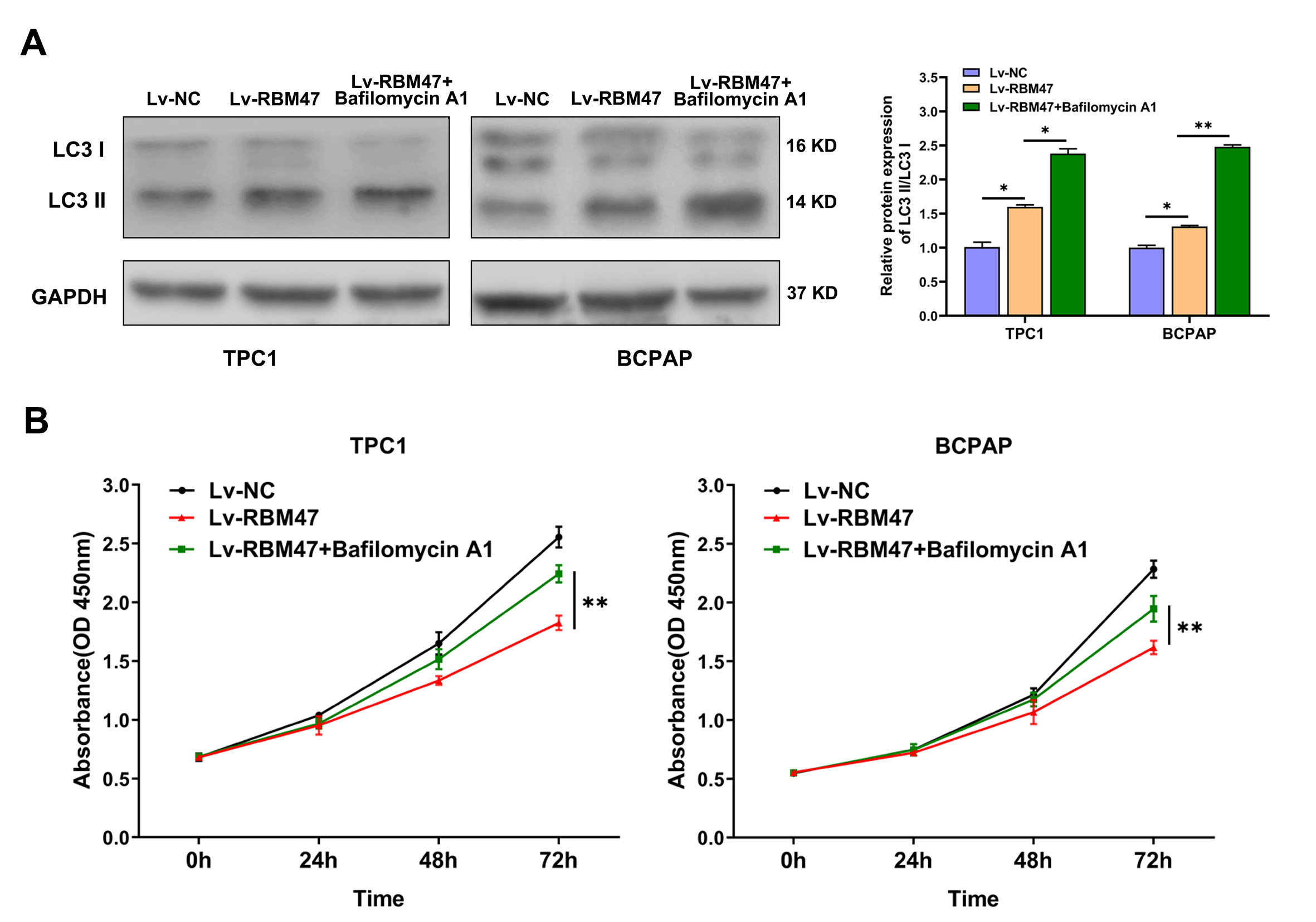

Supplement: Supplementary file 7 — Supplemental Figure1 [file 41419_2022_4728_MOESM7_ESM.jpg]

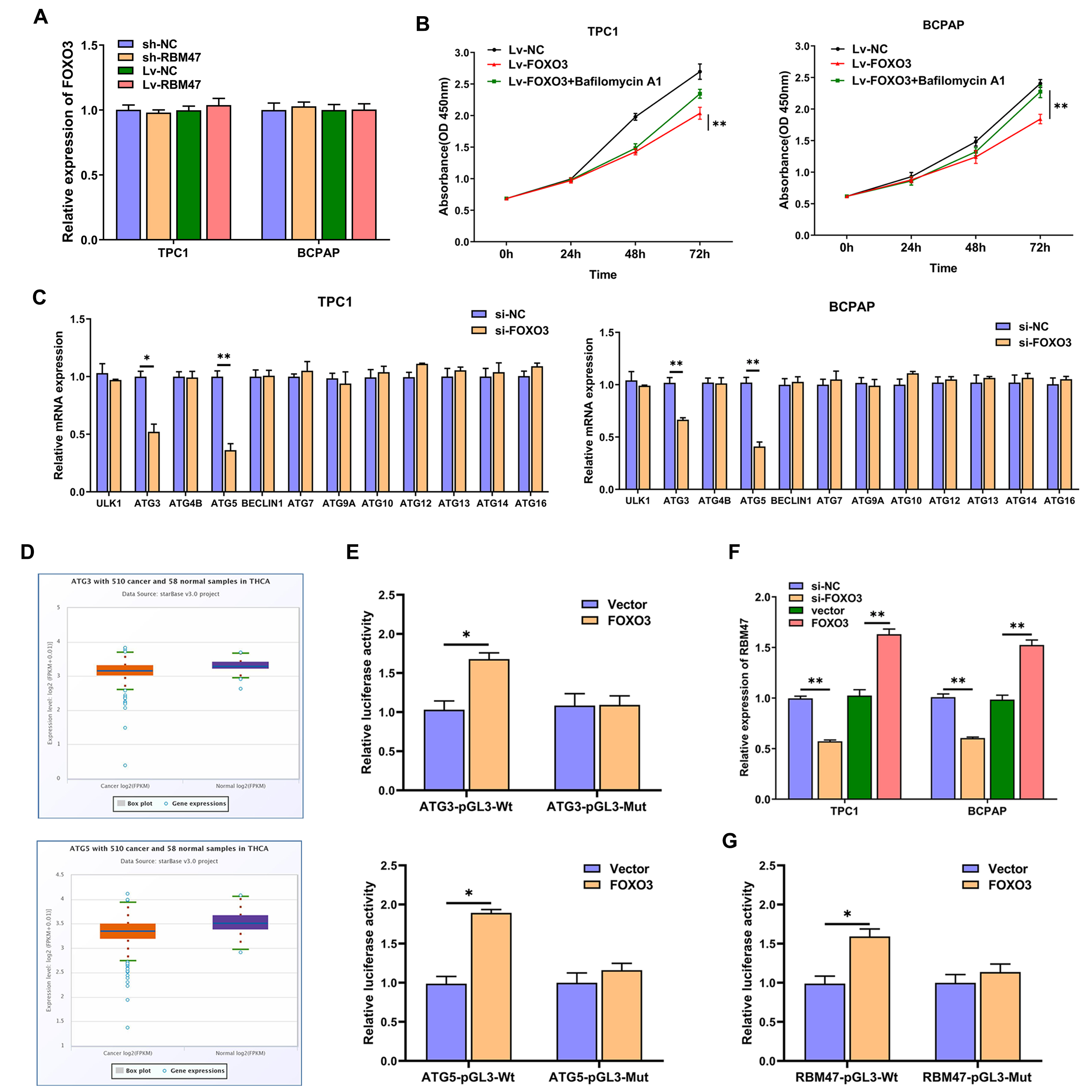

Supplement: Supplementary file 8 — Supplemental Figure2 [file 41419_2022_4728_MOESM8_ESM.jpg]

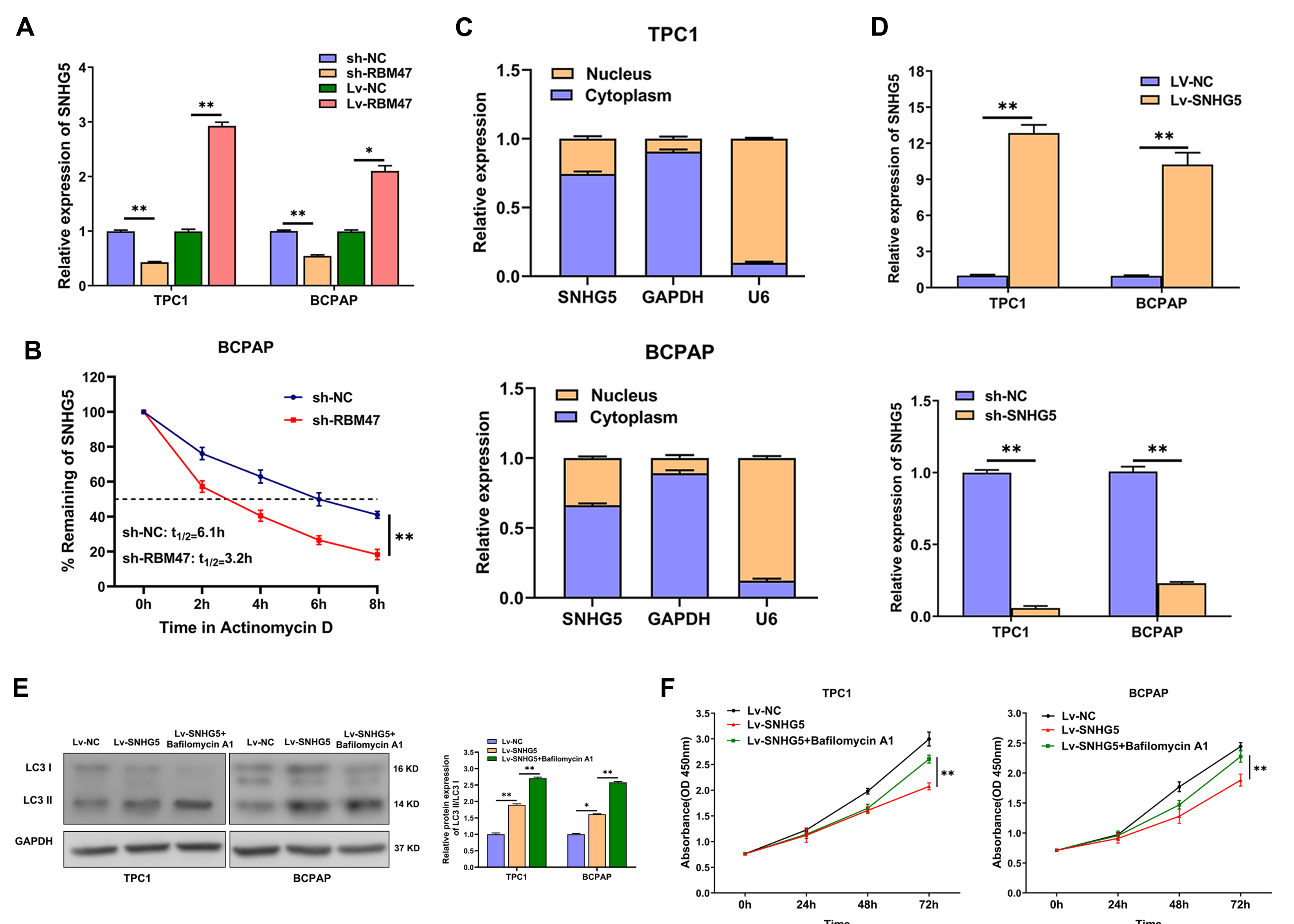

Supplement: Supplementary file 9 — Supplemental Figure3 [file 41419_2022_4728_MOESM9_ESM.jpg]
