# Supplementary material for: RBM47/SNHG5/FOXO3 axis activates autophagy and inhibits cell proliferation in papillary thyroid carcinoma
Source: Cell Death Dis. 2022 Mar 25;13(3):270. doi: 10.1038/s41419-022-04728-6 (PMC8956740; doi:10.1038/s41419-022-04728-6)
Supplement: Supplementary file 2 — Supplemental Figure legends [file 41419_2022_4728_MOESM2_ESM.docx]

**Figure S1 (A)** LC3 II/LC3 I ratio detected by WB in PTC cells with RBM47 upregulation and Bafilomycin A1 treated. **(B)** Proliferation evaluated by CCK-8 assay in PTC cells with RBM47 upregulation and Bafilomycin A1 treated. Statistical differences were analyzed using the independent samples t-test; data are shown as the mean ± standard error of the mean based on three independent experiments. *p < 0.05, **p < 0.01.

**Figure S2** **(A)** Relative mRNA levels of FOXO3 were detected after RBM47 knockdown or upregulation. **(B)** Proliferation evaluated by CCK-8 assay in PTC cells with FOXO3 upregulation and Bafilomycin A1 treated. **(C)** The relative expression level of related ATGs in PTC cells with FOXO3 downregulation detected by qRT-PCR. **(D)** ATG3 and ATG5 expression in PTC tissues and normal tissues from TCGA database. **(E)** The luciferase reporter activity of ATG3 or ATG5 promoter in TPC1 cells overexpressing empty vector and FOXO3. **(F)** The relative mRNA levels of RBM47 in PTC cells with FOXO3 knockdown or upregulation. **(G)** The luciferase reporter activity of RBM47 promoter in TPC1 cells overexpressing empty vector and FOXO3. Statistical differences were analyzed using the independent samples t-test; data are shown as the mean ± standard error of the mean based on three independent experiments. *p < 0.05, **p < 0.01.

**Figure S3** **(A)** The relative mRNA expression of SNHG5 in PTC cells with RBM47 knockdown or upregulation. **(B)** The half-life of SNHG5 in BCPAP cells treated with RBM47 knockdown in BCPAP cells. **(C)** The nuclear and cytoplasmic RNA fractions isolated from PTC cells. The cytoplasm location of SNHG5; GAPDH and U6 were used as controls. **(D)** The transfection efficiency of sh-SNHG5 and Lv-SNHG5 assessed by qRT-PCR in PTC cells. **(E)** LC3 II/LC3 I ratio detected by WB in PTC cells with SNHG5 upregulation and Bafilomycin A1 treated. **(F)** Proliferation evaluated by CCK-8 assay in PTC cells with SNHG5 upregulation and Bafilomycin A1 treated. Statistical differences were analyzed using the independent samples t-test; data are shown as the mean ± standard error of the mean based on three independent experiments. *p < 0.05, **p < 0.01.
