# Supplementary material for: RBM47/SNHG5/FOXO3 axis activates autophagy and inhibits cell proliferation in papillary thyroid carcinoma
Source: Cell Death Dis. 2022 Mar 25;13(3):270. doi: 10.1038/s41419-022-04728-6 (PMC8956740; doi:10.1038/s41419-022-04728-6)
Supplement: Supplementary file 3 — Supplementary Table 1 [file 41419_2022_4728_MOESM3_ESM.docx]

| Characteristics | n | High expression  (%) | Low expression  (%) | P |
| --- | --- | --- | --- | --- |
| Gender |  |  |  |  |
| Male | 28 | 13 (46.4) | 15 (53.6) | 0.656 |
| Female | 72 | 37 (51.3) | 35 (48.7) |  |
| Age (years) |  |  |  |  |
| <55 | 64 | 33 (51.6) | 31 (48.4) | 0.676 |
| ≥55 | 36 | 17 (47.2) | 19 (52.8) |  |
| Extrathyroidal extension |  |  |  |  |
| Yes | 22 | 10 (45.5) | 12 (54.5) | 0.629 |
| No | 78 | 40 (51.3) | 38 (48.7) |  |
| TNM staging |  |  |  |  |
| I–II | 74 | 42 (56.8) | 32 (43.2) | **0.022*** |
| III–IV | 26 | 8 (30.8) | 18 (69.2) |  |
| Lymph node metastasis |  |  |  |  |
| Yes | 86 | 41 (47.7) | 45 (52.3) | 0.249 |
| No | 14 | 9 (64.3) | 5 (35.7) |  |
| Multifocality |  |  |  |  |
| Yes | 19 | 11 (57.9) | 8 (42.1) | 0.444 |
| No | 81 | 39 (48.2) | 42 (51.8) |  |
| Tumor size (cm) |  |  |  |  |
| <2 | 66 | 42 (63.6) | 24 (36.4) | **<0.01*** |
| ≥2 | 34 | 8 (23.5) | 26 (76.5) |  |
| Hashimoto thyroiditis |  |  |  |  |
| Yes | 15 | 8 (53.3) | 7 (46.7) | 0.779 |
| No | 85 | 42 (49.4) | 43 (50.6) |  |

**Table S1 Correlation between RBM47 expression and clinicopathological features in papillary thyroid cancer (PTC) (n = 100)**
