# Supplementary material for: RBM47/SNHG5/FOXO3 axis activates autophagy and inhibits cell proliferation in papillary thyroid carcinoma
Source: Cell Death Dis. 2022 Mar 25;13(3):270. doi: 10.1038/s41419-022-04728-6 (PMC8956740; doi:10.1038/s41419-022-04728-6)
Supplement: Supplementary file 4 — Supplementary Table 2 [file 41419_2022_4728_MOESM4_ESM.docx]

| Characteristics | n | High expression  (%) | Low expression  (%) | P |
| --- | --- | --- | --- | --- |
| Gender |  |  |  |  |
| Male | 28 | 10 (35.7) | 18 (64.3) | 0.125 |
| Female | 72 | 38 (52.8) | 34 (47.2) |  |
| Age (years) |  |  |  |  |
| <55 | 64 | 29 (45.3) | 35 (54.7) | 0.473 |
| ≥55 | 36 | 19 (52.8) | 17 (47.2) |  |
| Extrathyroidal extension |  |  |  |  |
| Yes | 22 | 8 (36.4) | 14 (63.6) | 0.216 |
| No | 78 | 40 (51.3) | 38 (48.7) |  |
| TNM staging |  |  |  |  |
| I–II | 74 | 36 (48.6) | 38 (51.4) | 0.826 |
| III–IV | 26 | 12 (46.1) | 14 (53.9) |  |
| Lymph node metastasis |  |  |  |  |
| Yes | 86 | 37 (43.1) | 49 (56.9) | **0.013*** |
| No | 14 | 11 (78.6) | 3 (21.4) |  |
| Multifocality |  |  |  |  |
| Yes | 19 | 10 (52.6) | 9 (47.4) | 0.653 |
| No | 81 | 38 (46.9) | 43 (53.1) |  |
| Tumor size (cm) |  |  |  |  |
| <2 | 66 | 37 (56.1) | 39 (43.9) | **0.024*** |
| ≥2 | 34 | 11 (32.4) | 23 (67.6) |  |
| Hashimoto thyroiditis |  |  |  |  |
| Yes | 15 | 10 (66.7) | 5 (33.3) | 0.116 |
| No | 85 | 38 (44.7) | 47 (55.3) |  |

**Table S2 Correlation between SNHG5 expression and clinicopathological**

**features in papillary thyroid cancer(PTC) (n = 100)**
