# Supplementary material for: RBM47/SNHG5/FOXO3 axis activates autophagy and inhibits cell proliferation in papillary thyroid carcinoma
Source: Cell Death Dis. 2022 Mar 25;13(3):270. doi: 10.1038/s41419-022-04728-6 (PMC8956740; doi:10.1038/s41419-022-04728-6)
Supplement: Supplementary file 5 — Supplementary Table 3 [file 41419_2022_4728_MOESM5_ESM.docx]

**Table S3. Primers and siRNA sequences used in this paper**

| **Primer** | **Sense (5′–3′)** | **Antisense (5′–3′)** |
| --- | --- | --- |
| RBM47 | ATCAGCAATCCTTGGCTCAC | CCTTGGGATTCCTCTGTTCA |
| SNHG5 | CTGGGCGGGTGGTAGGAACAAT | TTCACTGGCTACTCGTCCACACTCA |
| FOXO3 | GAAGAACTCCATCCGGCACA | GCTCTTGCCAGTTCCCTCAT |
| ATG3 | GACCCCGGTCCTCAAGGAA | TGTAGCCCATTGCCATGTTGG |
| ATG5 | AATCAGGTTTGGTGGAGGCA | CAGTGGAGGAAAGCAGAGGTG |
| USP21 | CAGGTCTGCCTGATGAACGG | GCTAAGTTGGTCCGAGATGGG |
| GAPDH | GCACCGTCAAGGCTGAGAAC | TGGTGAAGACGCCAGTGGA |
|  |  |  |
| **Primer for CHIP** | **Sense (5′–3′)** | **Antisense (5′–3′)** |
| ATG3-primer 1 | CCGGCCTCGCTTTTACTCTT | GGGGTTTTAGTGTCCTCAGCC |
| ATG3-primer 2 | TGCGACTTGAGCTTAAAGCA | TTGCGTTTAGGGTCTGCCAA |
| ATG3-primer 3 | ACAGAACCAAAGGCAAGAGAGA | TCTGCCAACAAGGTCGTTCA |
| ATG5-primer 1 | CTGCGGTGGTTCCAACAAAG | ACTGCCTCCCTAGAGCTTGA |
| ATG5-primer 2 | AGAGGACCATAACTGTGGCT | TCAAGTTAGGGAAGCATGCC |
| ATG5-primer 3 | TGTGCCTGGTCCCAACATTT | GCCTTCCCAGTGTCAGACAA |
| RBM47-primer 1 | GTGACAGAGCGAGAATCCGT | CGGGCCTCCCTTTTTCTCTT |
| RBM47-primer 2 | AATGGCTCAGTGACCTTGGG | GCTGGTCTCGAACTCCTGAC |
| RBM47-primer 3 | GGTGTGGTGGTGCATGACTA | GCGTGTCCTCAAATGCCATC |
| RBM47-primer 4 | GGAACCCACAGACACTGAGG | CCCGACCCTCAACTCCTTTC |
|  |  |  |
| **siRNA or shRNA** | **Sense (5′–3′)** | **Antisense (5′–3′)** |
| SNHG5 shRNA | CAGTGAAGATAATGAATGTTT |  |
| RBM47 shRNA | CACGGTGGCTCCAAACGTTCA |  |
| FOXO3 siRNA | CAAGCACAGAGUUGGAUGATT |  |
| USP21 siRNA | CUGUGAAGCCCUUUAAACA |  |
