# Supplementary material for: RBM47/SNHG5/FOXO3 axis activates autophagy and inhibits cell proliferation in papillary thyroid carcinoma
Source: Cell Death Dis. 2022 Mar 25;13(3):270. doi: 10.1038/s41419-022-04728-6 (PMC8956740; doi:10.1038/s41419-022-04728-6)
Supplement: Supplementary file 6 — Supplementary Table 4 [file 41419_2022_4728_MOESM6_ESM.docx]

**Table S4. Information of the antibodies and inhibitors used in this paper**

| Antibody | Brand | Concentration |  |
| --- | --- | --- | --- |
| RBM47 | Abcam, ab167164 | 1:1000 (WB) |  |
| FOXO3 | Abcam, ab109629 | 1:4000 (WB) |  |
| LC3 | Abcam, ab192890 | 1:2000 (WB) |  |
| USP21 | Proteintech, 17856-1-AP | 1:1000 (WB) |  |
| ATG3 | Abcam, ab108251 | 1:4000 (WB) |  |
| ATG5 | Abcam, ab108327 | 1:2500 (WB) |  |
| GAPDH | Proteintech, 10494-1-AP | 1:2000 (WB) |  |
| LC3 | Abcam, ab192890 | 1:500 (IF) |  |
| FOXO3 | Proteintech, 10849-1-AP | 1:200 (IF) |  |
| RBM47 | Abcam, ab167164 | 1:100 (IHC) |  |
| FOXO3 | Proteintech, 10849-1-AP | 1:100 (IHC) |  |
| USP21 | Proteintech, 17856-1-AP | 1:100 (IP) |  |
| FOXO3 | Proteintech, 10849-1-AP | 1:100 (IP) |  |
|  |  |  |  |
| Inhibitor | Brand | Concentration | Time |
| Bafilomycin A1 | Abcam, ab120497 | 20nM | 12h |
| Actinomycin D | MCE, HY-17559 | 2 μg/mL | 4-12h |
| Cycloheximide | MCE, HY-12320 | 100 ng/mL | 4-12h |
| MG 132 | MCE, HY-13259 | 10 mM | 12h |
